# Genetic differentiation in an endangered and strongly philopatric, migrant shorebird

**DOI:** 10.1186/s12862-021-01855-0

**Published:** 2021-06-19

**Authors:** Nelli Rönkä, Veli-Matti Pakanen, Angela Pauliny, Robert L. Thomson, Kimmo Nuotio, Hannes Pehlak, Ole Thorup, Petteri Lehikoinen, Antti Rönkä, Donald Blomqvist, Kari Koivula, Laura Kvist

**Affiliations:** 1 Ecology and Genetics Research Unit, University of Oulu, P.O. Box 3000, 90014 Oulu, Finland; 2 Department of Biological and Environmental Sciences, University of Gothenburg, P.O. Box 463, 405 30 Gothenburg, Sweden; 3 Section of Ecology, Department of Biology, University of Turku, 20014 Turku, Finland; 4 Percy FitzPatrick Institute of African Ornithology, DST-NRF Centre of Excellence, University of Cape Town, Rondebosch, 7701 South Africa; 5 Environmental Agency, Valtakatu 11, 28100 Pori, Finland; 6 Institute of Agricultural and Environmental Sciences, Estonian University of Life Sciences, Kreutzwaldi 5, 51014 Tartu, Estonia; 7 OÜ Xenus, Koguva, 94724 Muhu Island, Saare Estonia; 8 V. Vedsted Byvej 32, Vester Vedsted, 6760 Ribe, Denmark; 9 The Helsinki Lab of Ornithology, Finnish Museum of Natural History, University of Helsinki, 00014 Helsinki, Finland

**Keywords:** Fragmentation, Dispersal, Microsatellites, Population structure, Genetic variation, *Calidris alpina schinzii*, Baltic Sea

## Abstract

**Background:**

Populations living in fragmented habitats may suffer from loss of genetic variation and reduced between-patch dispersal, which are processes that can result in genetic differentiation. This occurs frequently in species with reduced mobility, whereas genetic differentiation is less common among mobile species such as migratory birds. The high dispersal capacity in the latter species usually allows for gene flow even in fragmented landscapes. However, strongly philopatric behaviour can reinforce relative isolation and the degree of genetic differentiation. The Southern Dunlin (*Calidris alpina schinzii*) is a philopatric, long-distance migratory shorebird and shows reduced dispersal between isolated breeding patches. The endangered population of the Southern Dunlin breeding at the Baltic Sea has suffered from habitat deterioration and fragmentation of coastal meadows. We sampled DNA across the entire population and used 12 polymorphic microsatellite loci to examine whether the environmental changes have resulted in genetic structuring and loss of variation.

**Results:**

We found a pattern of isolation-by-distance across the whole Baltic population and genetic differentiation between local populations, even within the southern Baltic. Observed heterozygosity was lower than expected throughout the range and internal relatedness values were positive indicating inbreeding.

**Conclusions:**

Our results provide long-term, empirical evidence for the theoretically expected links between habitat fragmentation, population subdivision, and gene flow. They also demonstrate a rare case of genetic differentiation between populations of a long-distance migratory species. The Baltic Southern Dunlin differs from many related shorebird species that show near panmixia, reflecting its philopatric life history and the reduced connectivity of its breeding patches. The results have important implications as they suggest that reduced connectivity of breeding habitats can threaten even long-distance migrants if they show strong philopatry during breeding. The Baltic Southern Dunlin warrants urgent conservation efforts that increase functional connectivity and gene flow between breeding areas.

**Supplementary Information:**

The online version contains supplementary material available at 10.1186/s12862-021-01855-0.

## Background

Habitat fragmentation leads to small local populations that are susceptible to stochasticity [1, 2]. In such populations, genetic drift and inbreeding are expected to enhance population differentiation and reduce genetic variation, leading to increased homozygosity and the risk of fixation of slightly harmful alleles which, in turn, decreases population viability [3–5]. Inbreeding depression, and in the long-term genetic stochasticity due to drift, poses a substantial threat in isolated and rapidly declining populations (e.g. [1]), as shown by many case studies (e.g. [6–8]). For such populations, gene flow and connectivity to other populations are crucial for maintaining genetic variation [9, 10].

The effects of a small population size may be reinforced by life-history characteristics such as poor dispersal ability or philopatry. Strong natal and adult philopatry suggests that there must be benefits from returning to the natal or previous breeding site [11]. However, philopatry can be a detrimental strategy at the population level, when fragmentation-driven reduction in connectivity has reduced gene flow [12]. Strong philopatry is expected to lead to genetic structuring, isolation-by-distance (IBD) and increased inbreeding due to decreased opportunities for individuals to mate outside of kin [2]. Multiple examples of these processes exist in species with reduced mobility, but they are much less common in birds, especially in long-distance migratory species, because of their higher dispersal rates [13, 14].

The Southern Dunlin (*Calidris alpina schinzii*) is a small, migratory shorebird species breeding exclusively in short-vegetated and wet grasslands [15, 16]. The Baltic population, which breeds mainly on coastal grasslands, has suffered a dramatic decline (about 60% during the last 20 years) and is one of the most endangered shorebird populations in Europe [17, 18] despite the species-level assessment of “Least Concern” [18]. In the beginning of the twentieth century, the Southern Dunlin was common and widespread in most parts of the Baltic [19]. By the start of the twenty-first century, the population size of the Baltic Southern Dunlin had declined to 1110–1360 breeding pairs [17]. Since then, several local populations have gone extinct, and the number of pairs is currently closer to 500 pairs [18]. While the populations have suffered from high nest predation pressure across the range [16, 20, 21] and possibly decreased adult survival [22], the initial reasons for the decline were likely large-scale agricultural changes and eutrophication which led to overgrowth, habitat loss, and fragmentation of previously connected meadow systems [18]. It is important to note that the population declines have continued despite breeding habitats being available in many areas around the Baltic (see [7]).

Habitat fragmentation can be expected to lead to genetic effects in the Southern Dunlin because both adults [23–25] and juveniles [26] are highly site-faithful to their breeding and natal sites. Yet, an analysis using data collected mostly before the drastic declines occurring after the turn of the twenty-first century, detected no genetic structuring or signs of genetic impoverishment in Baltic Southern Dunlins [27]. However, because reduced structural connectivity of patches has been shown to lead to decreased between-patch movements in this species [26], the current situation differs from the historical situation when breeding sites of the Southern Dunlin around the Baltic Sea were better connected and environmental predictability, favoring philopatry, was probably higher. Therefore, habitat fragmentation has since likely reduced the movements of individuals between populations. The resulting reduction in gene flow has already led to inbreeding, substantially increasing the likelihood of extinction of some populations [7]. Given these findings and the incessant decline of the Baltic population, we expected an overall reduction in genetic variation as well as increased genetic differentiation – even in this long-distance migratory species.

Building on extensive sampling from the entire Baltic Southern Dunlin population and genetic analyses based on polymorphic microsatellite markers, we (A) examine whether the populations at the Baltic are genetically differentiated, and (B) estimate levels of genetic variation of the Baltic populations in order to assess if the observed decline, increased isolation, and lack of connectivity of breeding sites have had an effect since previous studies. Importantly, the genetic connectedness of the Bothnian Bay population at the northernmost location of the Baltic Sea in Finland [28] to the *schinzii* populations in the southern Baltic and the *alpina* population in Lapland has never been studied. Therefore, we further (C) examine whether there is indication of gene flow between the nominate subspecies *C*. *a*. *alpina* and Baltic Southern Dunlin populations with special emphasis on the population at Bothnian Bay. Finally, we (D) discuss the evolutionary implications of our study and suggest relevant conservation measures.

## Results

### Genetic diversity

Genotyping error rate was low, with a mean of 1.6% when genotyped twice. When the ambiguous samples were again genotyped twice, the error rate dropped to 0.1% (only one locus in four individuals remained unclear—this data were excluded from the analyses). In two populations, the presence of null alleles was suggested for loci CAS23 and Cme1 (see Additional file 1: Table S1 for information on the loci used), and these loci were also suspected to show stuttering in other populations. For CAS23, however, this was likely caused by the marker being sex-linked. Since the error rate was low and the suggested genotyping errors were not consistent among populations, all loci were used in the analyses. Linkage disequilibrium was present only in a few locus pairs, seemingly randomly in different populations, suggesting no strong linkage between the loci.

Allelic richness (A), corrected for the difference in sample size, was similar in all *schinzii* populations (varying from 3.61 in Pori to 3.76 in Jurmo; Table 1). The highest *A* (3.93) was found in the *alpina* subspecies. Observed heterozygosity was lower than expected (i.e. F_IS_ values were positive) in every population (Table 1). F_IS_ was significant in Pori and Jurmo, and especially in Estonia. The lowest F_IS_ was in Bothnian Bay (0.001). The mean internal relatedness (IR) values per population were highest in Pori (0.231), Denmark (0.133) and Estonia (0.093), and lowest in Bothnian Bay (0.020; Table 1).

**Table 1:** Population and sample size, genetic diversity and relatedness estimates for the Baltic Southern Dunlin (*Calidris alpina schinzii*) populations and Dunlins (*C. a. alpina*) from Finnish Lapland

Population	Pair number	N	A	H_O_	H_E_	F_IS_	IR
Bothnian Bay	50	196	3.69	0.756	0.757	0.001	0.020
Pori	5	8	3.61	0.635	0.777	0.138*	0.231
Jurmo	4	6	3.76	0.678	0.800	0.126*	0.086
Estonia	200	53	3.75	0.689	0.767	0.082**	0.093
Denmark	170	4	3.69	0.720	0.787	0.097	0.133
Western Sweden	7	30	3.66	0.736	0.759	0.031	0.073
Eastern Sweden	84	26	3.74	0.731	0.768	0.049	0.069
Southern Sweden	18	25	3.71	0.738	0.761	0.030	0.059
*C. a. alpina*	Not known	26	3.93	0.748	0.795	0.058	0.051

*N*  Number of samples, *A * Allelic richness, *H*_*O*_  Observed heterozygosity, *H*_*E*_ Expected heterozygosity, *F*_*IS*_ Observed heterozygosity relative to the heterozygosity expected under random mating [1], IR = Internal relatedness; *p < 0.05, **p < 0.001; Pair number indicates the estimated number of breeding pairs during the time of sampling. Estimates for the Swedish sites were taken from Flodin et al. [29]

### Isolation-by-distance

**Fig. 1:**
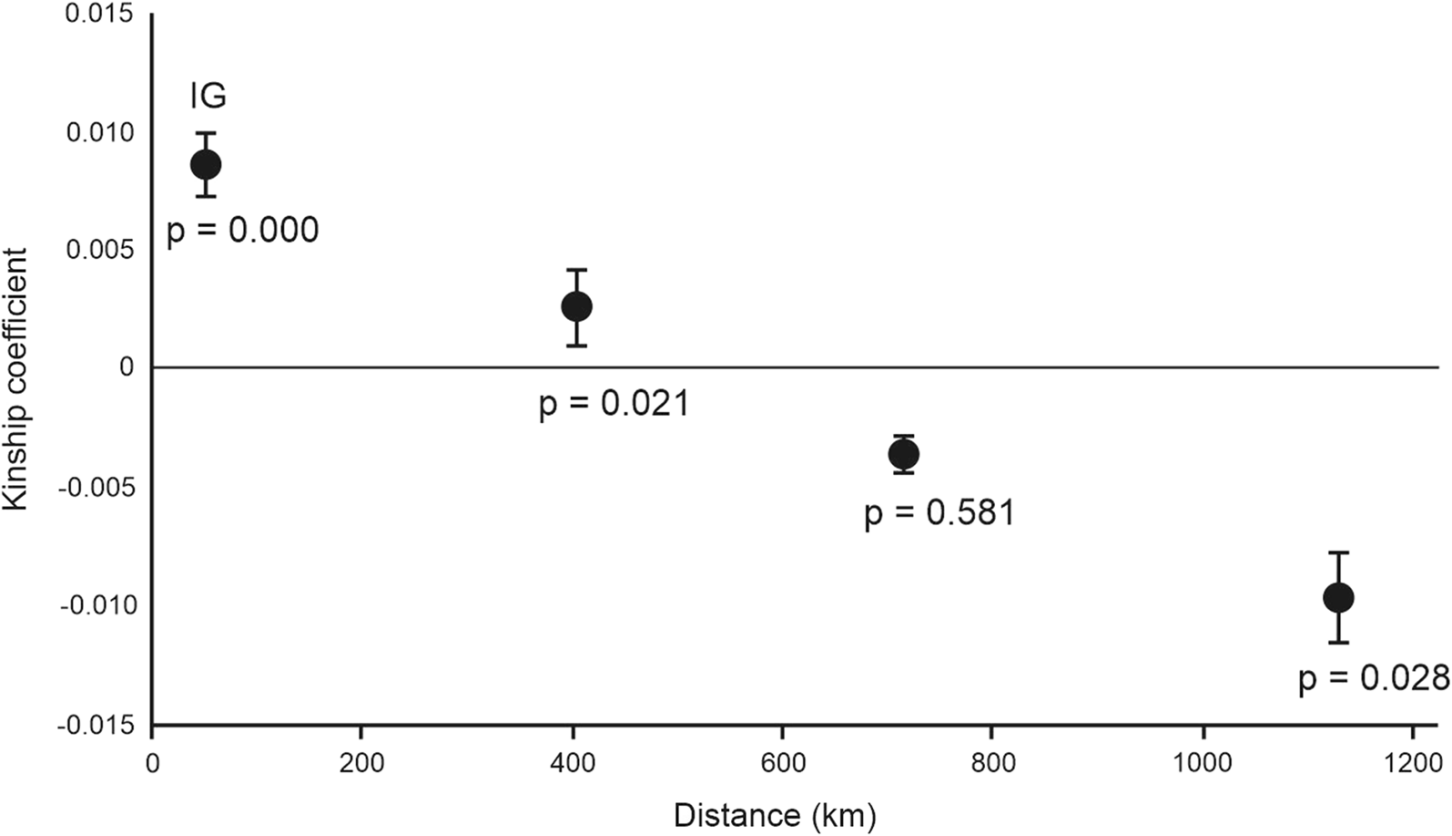
Mean kinship coefficient (genetic similarity) versus the mean distances (km) of three distance classes and the intra-group class, IG, of the Baltic Southern Dunlin (*Calidris alpina schinzii*). Whiskers indicate standard error

We found that the kinship coefficient slowly decreased with increasing distance between individuals, indicating an isolation-by-distance pattern (Fig. 1) with regression coefficient per one km being -0.00002 (SE = 0.000004; *p* = 0.021, number of individuals = 374). The intra-group (IG) class and the first distance class (mean distance 405 km) showed significantly positive kinship coefficients (*p* = 0.000 and *p* = 0.021, respectively), whereas the third and fourth distance classes (mean distances 716 and 1129 km, respectively) were negative, with the last class significantly so (*p* = 0.028).

### Genetic structure

**Fig. 2:**
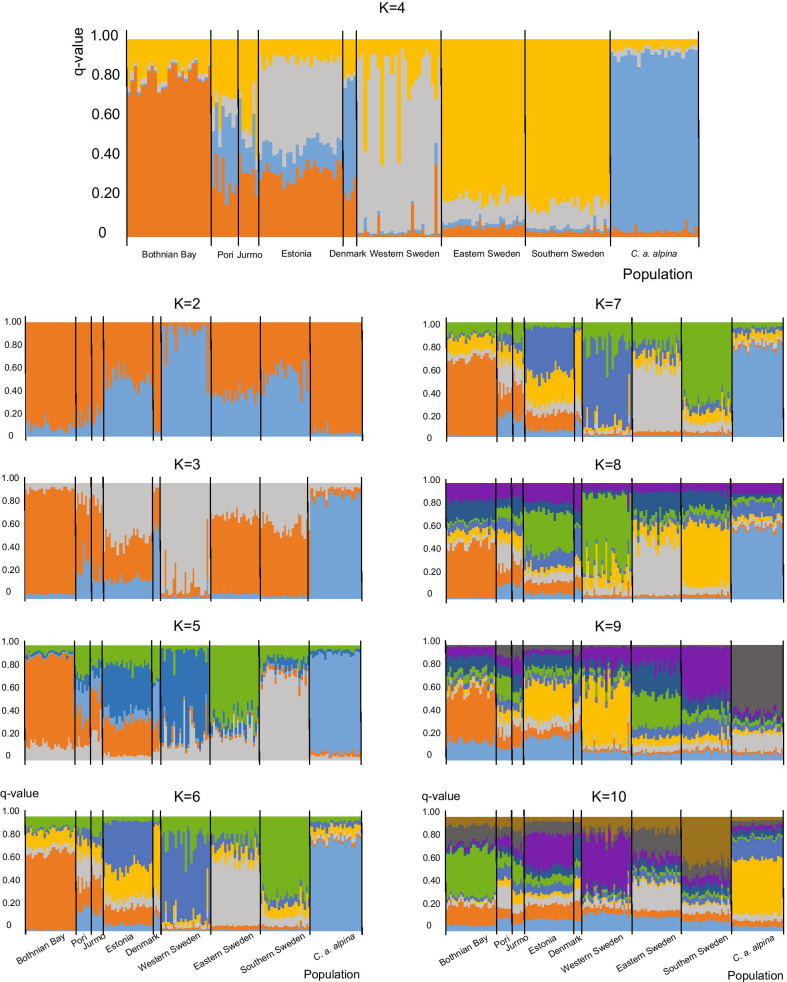
Structure results for the Baltic Southern Dunlin (*Calidris alpina schinzii*) populations and Dunlins (*C. a. alpina*) from Finnish Lapland. Bar plots with different values of K (2–10). The highest ΔK value was at K = 4 (see Additional file 2: Figure S1). Each individual is represented by a vertical bar divided into four differently colored segments, where the amount of each color indicates the proportional probability of belonging to each inferred cluster

**Fig. 3:**
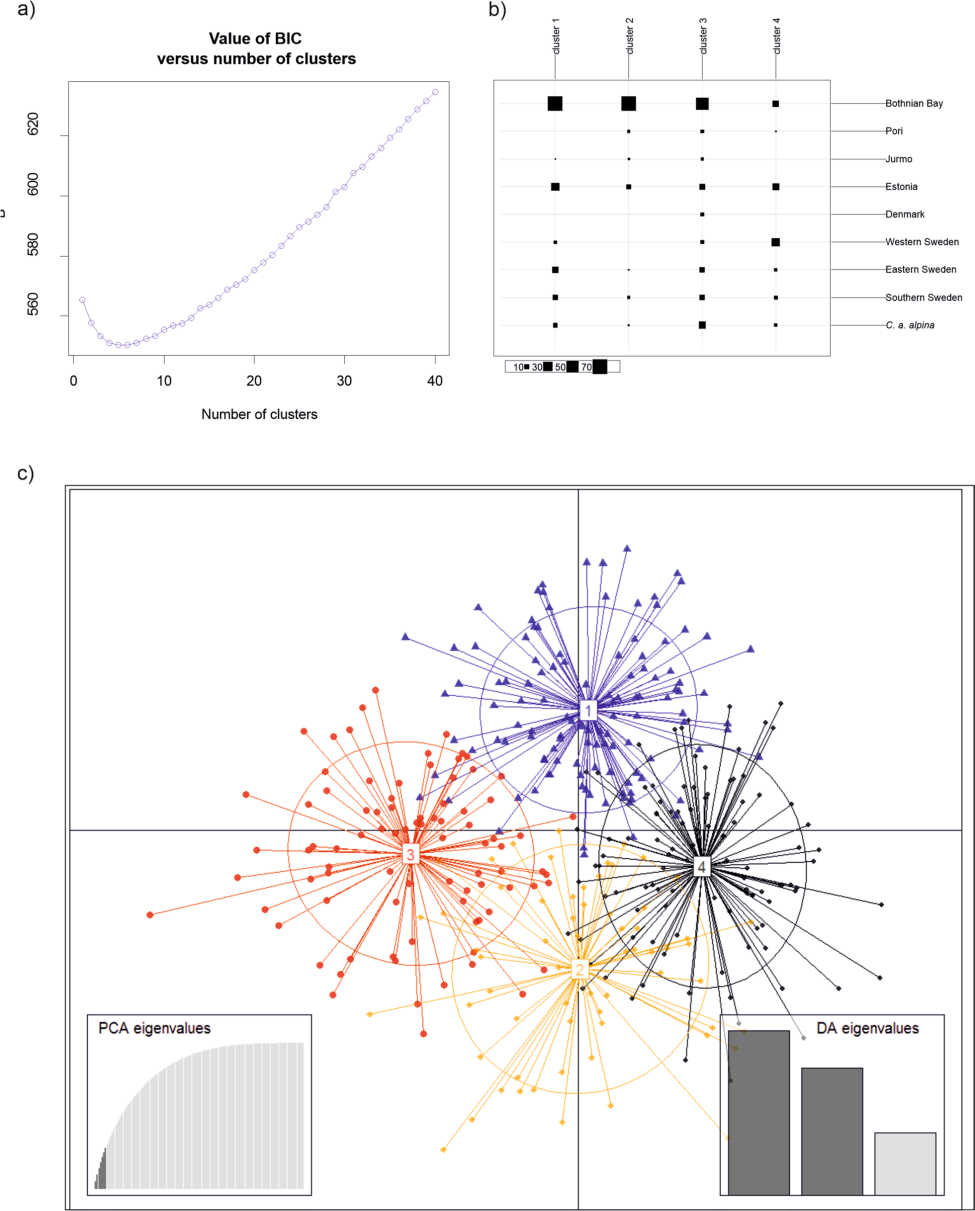
DAPC results. **a** A graph of BIC values for K 1–40, **b** the memberships of individuals of each study population to each cluster for K = 4, and **c** a scatterplot of individuals for K = 4

The clustering analysis with program Structure resulted in the highest ΔK value at K = 4 (Additional file 2: Figure S1 and Table S2). Birds from the *alpina* population formed their own cluster (Fig. 2 upper panel), with *q*-values of 0.835–0.932. Most individuals from the Bothnian Bay were assigned to one cluster (*q*-values 0.715–0.883), with the remaining individuals mainly showing similarity with the eastern/southern Swedish cluster (*q*-values 0.092–0.248). The populations at Pori and Jurmo were similar to the Estonian population, with mixed ancestry consisting for the most part of clusters found in Bothnian Bay and eastern/southern Sweden. Estonian individuals also showed mixed ancestry, mostly with the western Swedish (*q*-values 0.360–0.563) and Bothnian Bay clusters (*q*-values 0.245–0.391). Individuals from eastern and southern Sweden clustered together (*q*-values 0.738–0.892), and both showed some ancestry with the western Swedish cluster. Western Sweden mostly formed its own cluster (*q*-values 0.584–0.939), but a few individuals had probabilities below 0.400 of belonging to that cluster, instead indicating partial membership with the eastern/southern Swedish cluster. The few individuals from Denmark were similar to the Pori and Jurmo populations, but with a higher proportion of their genotypes assigned to the *alpina* cluster (*q* = 0.217–0.603) with K = 4. However, they seemed to form a separate genetic constitution with higher K values (Fig. 2). BIC values for the best number of clusters detected with DAPC resulted in very similar values for K 4–6 (K = 4: BIC = 550.980, K = 5: BIC = 550.261, K = 6: BIC = 550.265; Fig. 3a). As the clearest drop was from K = 3 (553.415) to K = 4, we chose K = 4. The resulting memberships of individuals to each cluster and a scatterplot are shown in Fig. 3b, c. Comparing these results with those from Structure, the number of clusters was similar but the geographic pattern was less clear.

F_ST_ values between populations varied from -0.043 to 0.084 (Table 2), the overall among-population fixation index being highly significant (F_ST_ = 0.011, *p* < 0.001). The Bothnian Bay population differed from all Swedish and the Danish population, the Danish population from western and southern Sweden, and *alpina* from every other population except Pori, Jurmo and Estonia, which did not differ significantly from any population. The largest pairwise differences arose in pairwise D_est_ comparisons: they were up to an order of magnitude higher than, but still concordant with the F_ST_ values (*R*^2^ = 0.747, *p* < 0.001), varying from − 0.007 to 0.334 (Table 2).

**Table 2:** Population pairwise F_ST_ values below the diagonal and D_est_ values above it for the Baltic Southern Dunlin (*Calidris alpina schinzii*) populations and Dunlins (*C. a. alpina)* from Finnish Lapland estimated from microsatellite data

	Bothnian Bay	Pori	Jurmo	Estonia	Denmark	Western Sweden	Eastern Sweden	Southern Sweden	*C. a. alpina*
Bothnian Bay		0.110^*^	0.020	0.070^*^	0.271^*^	0.157^*^	0.094^*^	0.127^*^	0.150^*^
Pori	− 0.022		0.030	0.053^NA^	0.247^NA^	0.135^*^	0.114	0.127^*^	0.096
Jurmo	− 0.023	− 0.043		− 0.007^NA^	0.208*	0.109 ^*^	0.008	0.032	0.029
Estonia	− 0.020	0.018	− 0.038		0.236^NA^	0.080^*^	0.071^*^	0.119^*^	0.100^*^
Denmark	0.075*	0.033	0.056	0.038		0.334^*^	0.216 ^NA^	0.268^*^	0.211^*^
Western Sweden	0.041*	− 0.011	0.006	− 0.026	0.084*		0.117^*^	0.130^*^	0.185^*^
Eastern Sweden	0.018*	− 0.028	-0.015	− 0.027	0.059	0.026^*^		0.088^*^	0.143^*^
Southern Sweden	0.026*	− 0.026	-0.017	− 0.021	0.071*	0.037^*^	0.022^*^		0.129^*^
*C. a. alpina*	0.034*	− 0.034	-0.014	− 0.012	0.067*	0.049^*^	0.035^*^	0.030^*^	

^*^p < 0.05; ^NA^ = significance could not be calculated due to sample size/missing data

## Discussion

We show that the Southern Dunlin populations breeding on the Baltic coastal meadows are genetically differentiated, providing a rare example of genetic structuring on a regional scale of a long-distance migratory species. This finding is in strong contrast to observations from many other northern shorebird species, which show limited genetic differentiation and high levels of gene flow on  a geographic scale similar to our study [30] and even across much larger spatial scales (e.g. [31–35]). Genetic differentiation has mainly been found in island populations of otherwise panmictic species (e.g. [36], but see also [37]). Indeed, the Southern Dunlin is a habitat specialist, and the fragmented habitat patches resemble islands amidst a matrix of unsuitable breeding habitat. Accordingly, genetic differentiation is partly linked to the distance between breeding sites, as indicated by the significant IBD pattern. While these results are in line with the continued fragmentation of its breeding range, the philopatric life history of the species may be the process that prevents movement of this otherwise mobile species and thus further reduces functional connectivity of the populations, eventually affecting allele frequencies and resulting into genetic differentiation.

The IBD pattern showed a decrease of genetic similarity with increasing geographic distance across the Baltic region. In general, individuals breeding up to 400 km away have significantly positive kinship coefficients (i.e. they are genetically more similar to each other than if chosen by random), which is consistent with known long-distance movements between Pori and Bothnian Bay [28]. Genetic similarity becomes negative (i.e. individuals are genetically less similar than if chosen by random) when the distance extends over 600 km. Accordingly, the highest F_ST_ values were observed between Bothnian Bay and the southwestern populations (Denmark and the Swedish populations) that are furthest apart. The Estonian population, which is located somewhat in the middle between the Bothnian Bay and the southwestern populations, showed mixed ancestry in the Structure analysis, mostly with the Bothnian Bay and western Swedish populations. This indicates that the Estonian population receives immigrants from other parts of the range. DAPC suggested the best number of clusters would be the same four as suggested by the Structure analysis. However, the clustering was not as clearly in accordance with the geographic origins of the samples as in Structure. Based on the results of these two analyses, it seems that there is a gradual change in allele frequencies along the Baltic Sea from the southwestern areas through Estonia towards the Bothnian Bay, resembling the cline reported by Marthinsen et al. [38] but on a much smaller spatial scale.

Importantly, we also found differentiation (both with fixation indices and clustering analyses) within a smaller scale of 200 to 500 km between the populations in the southern Baltic. This result fits earlier reports that show movements to be rare in these local populations [7, 24, 26]. Despite the relatively short distances to other populations in the southwestern Baltic, western Sweden does not receive immigrants [7]. Therefore, even though movement generally decreases with increasing distance between breeding sites [26], unknown environmental factors operating together with distance seem to create ecological barriers and differentiation also on smaller scales. It is, for example, possible that the breeding sites in western Sweden are located too far west from the migration route that follows the coast of the Baltic Sea [39].

We identified four genetic clusters within our data: three of them were formed by the Baltic Southern Dunlin individuals and the fourth by *C. a. alpina* individuals (with the exception of Danish Southern Dunlins that clustered with *alpina* at small K-values; Fig. 2). This contradicts earlier results by Marthinsen et al. [38], who could not distinguish between Dunlin subspecies with clustering analysis using seven microsatellites. Given our results, it seems unlikely that *C. a. alpina* mix with the Southern Dunlin populations as a result of migratory short-stopping, as indicated in other subspecies [40].

We found lower levels of heterozygosity than expected (i.e. positive F_IS_ values) and positive estimates of internal relatedness throughout the range, which is consistent with inbreeding. Indeed, previous work has documented severe inbreeding in the very small population in western Sweden [7] and a significantly positive inbreeding coefficient in the island of Öland, Eastern Sweden [27]. In the present study, the F_IS_ values of these populations were positive though not statistically significant (Table 1), which may be a sampling effect. On the other hand, the highly significant F_IS_ estimated for Estonia, one of the largest local populations, may in fact be explained by within-population genetic structure (Wahlund effect), which is further supported by the mixed ancestry in Estonia and weak differentiation from the other populations. The Bothnian Bay population showed the lowest F_IS_ and IR values and highest observed heterozygosity. These attributes are consistent with annually occurring immigration to the Bothnian Bay [28] and relatively high movement rates between breeding patches [26].

## Conclusions

Our results highlight that philopatry and reduced structural connectivity can result in population differentiation and IBD at a small spatial scale even in a mobile species. However, the pronounced site fidelity in the declining and fragmented Baltic Southern Dunlin population results in inbreeding ([7], this study) and may lead into an evolutionary trap [7, 41]. Indeed, the ongoing population decline suggests that the local populations across the Baltic are becoming smaller and more vulnerable to stochastic processes and eventually extinction. In addition to maintaining existing breeding sites, the Baltic Southern Dunlin therefore needs urgent conservation efforts that increase structural connectivity among present and potential breeding sites. For isolated and inbred populations such as the one in western Sweden, translocation of individuals might be the only way to ensure the exchange of genetic material. This could be achieved by captive breeding and introduction, perhaps from the genetically and geographically closest populations in eastern and southern Sweden.

## Methods

### Study species

The Southern Dunlin is one of several subspecies of Dunlin that breed across arctic and subarctic tundra, alpine wetlands, and wet grasslands in the temperate zone [43]. In addition to the Baltic region, Southern Dunlins breed in Iceland, British Isles, Faroe Islands and southeastern Greenland with an estimated 970 000–990 000 individuals [18, 44]. Their main autumn migration route follows the Atlantic coast of Europe and continues south to the main wintering areas in northwest and northern Africa, where the wintering grounds are shared with the northern subspecies *alpina* and *arctica* [39, 43, 45]. Based on previous genetic analyses, the Southern Dunlin is part of a larger phylogenetic group including its geographically closest subspecies *alpina* and *arctica*. Even though these subspecies are genetically very similar, some genetic differences have been found. These differences mainly reflect a gradual change in allele frequencies throughout their breeding ranges [38, 46, 47].

**Fig. 4:**
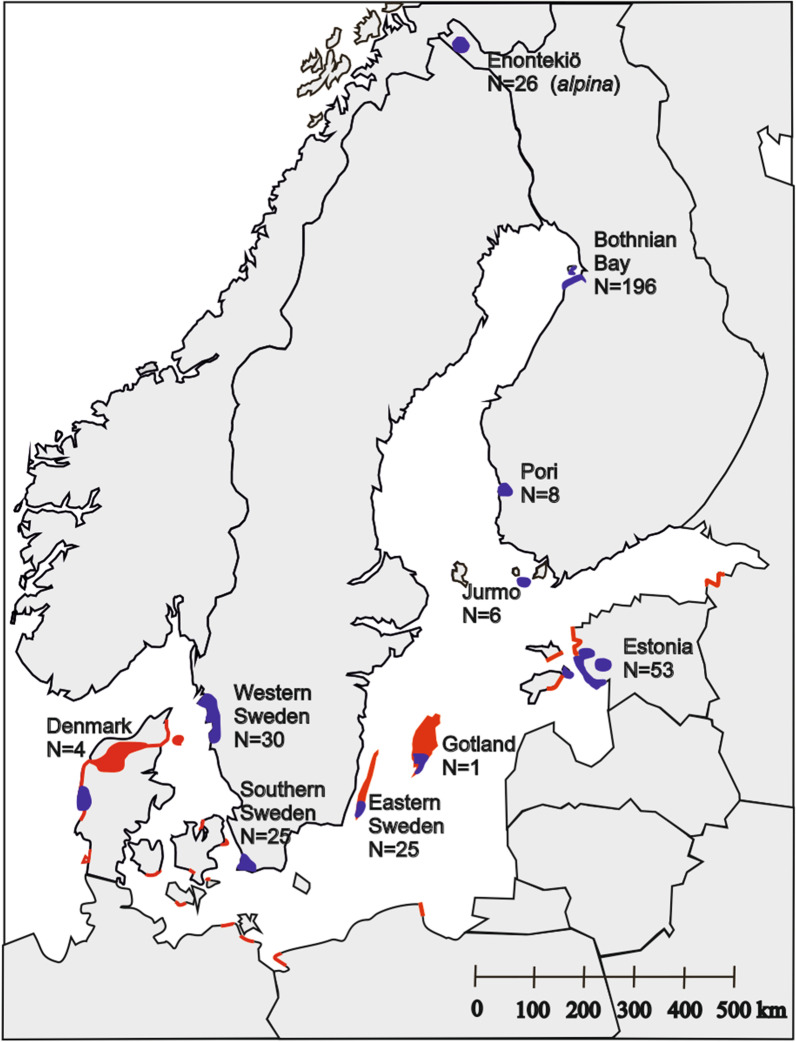
Locations of the sampled populations (in blue) of the Baltic Southern Dunlin (*Calidris alpina schinzii*) and Dunlin (*C. a. alpina*) with sample sizes (N) and the non-sampled (in red) breeding distribution of the Baltic Dunlin [18]. The breeding distribution was drawn according to [18] and Rönkä et al. (unpublished data). See text and [18] for details on the current distribution

The largest numbers of breeding Southern Dunlins in the Baltic area are found in Denmark and Estonia, where the populations are estimated to 170 and 180–230 pairs, respectively [18, 42, 48]. Sweden holds about 75 pairs [49] divided in three populations in western, eastern and southern Sweden, 250–300 km apart. Finland holds around 40–50 pairs (own observations). Most of them breed at Bothnian Bay; two other populations exist in southern Finland in Pori and Jurmo, over 400 km away (Fig. 4). The populations in Poland and Lithuania have disappeared and there is only one breeding site left in Germany [18].

### Sampling

Blood samples of 344 adult Southern Dunlins were collected from eight populations around the Baltic Sea during the breeding seasons in 1997–2016 (Fig. 4, Additional file 3: Table S3). The length of the sampling period varied among the populations; ranging from only 1 year in some to 17 years in western Sweden (Additional file 3: Table S3). However, even the longest sampling periods represent only a few generations given that the Dunlin has an average longevity of 7 years [18].

The samples were obtained by puncturing the brachial vein. In addition, four eggs with embryos from four different deserted nests were obtained from the Danish population. The only sample from Gotland was combined with the eastern Swedish population due to their proximity (< 150 km).

Samples (feathers) of the northern subspecies *alpina* were obtained from 26 breeding adults from Finnish Lapland, collected during 2010–2017. The total number of sampled Dunlins was thus 374.

### DNA extraction and microsatellite amplification

DNA was extracted from blood and tissue samples either with the standard phenol–chloroform method [50], or using the UltraClean® BloodSpin™ Kit or UltraClean® Tissue & Cells DNA Isolation Kit (MoBio Laboratories), and from feathers using the method described in Rönkä et al. [34]. Individuals were genotyped for 12 microsatellite loci, which were amplified in 10 µl volumes containing 20–100 ng of template DNA, 0.1 µM of each primer, 0.8–1 mM MgCl_2_, 0.2 mM of dNTPs, 1 µl of 10 × PCR-Buffer and 0.l U of DNA-polymerase (Biotools). The amplification profile was 94 °C for 1 min followed by 35 cycles of 94 °C for 30 s, 52–58 °C for 45 s (see Additional file 1: Table S1), 72 °C for 45 s and 72 °C for 10 min for final extension. The PCR reactions were run with ABI 3730 sequencer using GS500-Liz size standard (Applied Biosystems) and the loci were scored with GeneMapper v. 4.0. (Applied Biosystems), except for the Swedish samples, which were scored with CEQ^TM^8000 Genetic Analysis System (Beckman Coulter) using the Fragment Analysis Module v. 8.0.52. Due to possible differences between the allele sizes defined by the two sequencers, samples were calibrated by genotyping five Swedish individuals with both sequencers. Genotyping error rate was calculated by amplifying most individuals twice. If differences were found between the two runs, samples were genotyped twice more.

### Basic statistics

The microsatellite data were checked for potential genotyping errors (stutter bands, null-alleles and large allele dropouts) with MicroChecker v. 2.2.3. [51] for all populations except Denmark, Pori and Jurmo, which contained too few samples. GenePop v. 4.2. [52, 53] was used to test linkage disequilibrium (LD) and deviation from the Hardy–Weinberg (HW) equilibrium for each locus and population, as well as calculating population-wise F_IS_ (i.e. deviation from random mating). FSTAT v. 2.9.3.2. [54] was used to calculate allelic richness (individuals with any missing data excluded and using rarefraction to account for different sample sizes) and an Excel macro [55] to calculate internal relatedness of individuals. Observed and expected heterozygosity was calculated with Arlequin v. 3.5.1.2. [56] for each population. One locus (CAS23) was found to be sex-linked as females had always only one allele, whereas males were often heterozygous. This locus was excluded from the calculations of heterozygosity, F_IS_ and allelic richness. For the Structure run (see below), all females and the individuals whose sex remained undetermined were coded as both alleles missing regarding this locus.

### Isolation-by-distance

Isolation-by-distance within the Southern Dunlin was tested with program SpaGeDi v. 1.4. [57]. The program calculates the genetic relatedness of all possible pairs of individuals, compares the relatedness to the corresponding geographic distances and tests whether there is a correlation. The number and intervals of the distance classes were decided after fine-tuning them so that they met the ‘rule of thumb’ of SpaGeDi (# pairs > 500, % partic > 50% and CV partic < 1 per distance class; see SpaGeDi manual for further information). This resulted in three distance classes: birds breeding up to 650 km, 651–850 km and 851–1400 km from each other, respectively. In addition, an intra-group (i.e. population-wise) class was formed. The mean pairwise kinship coefficients of Loiselle et al. [58], an estimator especially suitable for loci with low-frequency alleles present, were estimated within each class. The mean kinship coefficients were plotted against the mean geographic distances of the classes. Significance was tested with 10 000 permutations and a jackknife procedure over loci was used to estimate standard errors for each distance class.

### Genetic population structure

An allele size permutation test [59] was performed with program SpaGeDi to test if stepwise mutations contribute to population structure. Observed R_ST_ values were compared to permuted ones (pR_ST_) using 10 000 random permutations. Observed values significantly larger than permuted ones indicate that stepwise mutations contribute to genetic structure, and R-statistics should be preferred over F-statistics [60]. As no significant differences were found (R_ST_ = 0.0300, pR_ST_ = 0.0261, *p* = 0.391), F-statistics were used in the analyses.

Genetic population structure was studied with program Structure v. 2.3.4. [61], which uses a Bayesian Markov Chain Monte Carlo (MCMC) approach to identify the number of genetically distinct clusters (K). The admixture model and correlated allele frequencies were used [62]. K was set from one to ten, and the program was run for 500 000 MCMC repeats with a burn-in of 50 000 and ten iterations for each K. Analyses were performed with the LOCPRIOR model [63], which takes the sampling locations into account. Uneven sampling can often lead to wrong inferences of hierarchical structure, as distinct populations with reduced sampling tend to be merged together, and individuals from extensively sampled populations can be split despite belonging to the same panmictic population [64]. The samples were therefore randomized to contain a maximum of 25 individuals per population. Program Structure Harvester [65] was used to summarize the cluster assignments across the iterations for each K and to estimate ΔK between the consecutive numbers of Ks using the method of Evanno et al. [66]; the highest ΔK should be the best estimator of the actual K. Program Clumpp [67] was used to obtain mean probabilities (*q*-values) of each individual belonging to the estimated K genetic clusters.

The genetic structure was further studied with the Discriminant Analysis of Principal Components (DAPC), a multivariate method for identifying genetically related individuals. DAPC was run with the package *adegenet* [68, 69] in R v. 7.5.1. [70]. Command find.clusters was used to detect the number of the genetic clusters in the data (K, from 1 to 40) and the DAPC was then performed with the most supported K. The command optim.a.score was used to find the best number of principal components retained and then the analysis was rerun with this number.

In addition, pairwise and overall F_ST_ values between the populations were calculated with program Arlequin [56]. Significance was estimated with 1 000 permutations and adjusted following the sequential Bonferroni method. In highly variable markers, genetic variation tends to be systematically underestimated when using fixation indices ([71, 72], but see also [73–75]). FST may approach zero even if the populations are strongly differentiated [72, 76] and it can also underestimate genetic differentiation when mutation rate is high relative to migration rate, as often is the case with microsatellites [7, 73]. A differentiation index, D_est_, accounts for small sample size and can be a better estimator of population differentiation than F_ST_ [72, 75, 76]. Therefore, we also calculated population pairwise D_est_ values using the DEMEtics package [77] in program R v. 3.5.2 [70]. The number of bootstrap replicates was set to 1 000, and the significance level was adjusted by sequential Bonferroni correction.

## Supplementary Information

**Additional file 1: Table S1.** Summary of microsatellites used in this study.

**Additional file 2: Figure S1.** ΔK values from the Structure analysis. **Table S2.** Mean LnPs and standard deviations for different K-values from program Structure Harvester.

**Additional file 3: Table S3.** Information on locations of sampled populations, sampling years and permits.

## Data Availability

The datasets generated and analysed during the current study are available in the Dryad repository [https://doi.org/10.5061/dryad.pk0p2ngnq].

## Abbreviations

A: Allelic richness
D_est_: A measure of genetic differentiation
F_IS_: Deviation from random mating
F_ST_: A measure of genetic differentiation
H_E_: Expected heterozygosity
H_O_: Observed heterozygosity
HW: Hardy–Weinberg equilibrium
IBD: Isolation-by-distance
IG: Intra-group
IR: Internal relatedness
K: Number of genetically distinct clusters
ΔK: Change in K
MCMC: Markov Chain Monte Carlo method
PCR: Polymerase chain reaction
R_ST_: A measure of genetic differentiation
pR_ST_: Permutated R_ST_ value

## References

[1] Keller LF, Waller DM (2002). Inbreeding effects in wild populations. Trends Ecol Evol.

[2] Frankham R, Ballou JD, Ralls K, Eldridge MDB, Dudash MR, Fenster CB (2017). Genetic management of fragmented animal and plant populations 2017.

[3] Hedrick PW, Kalinowski ST (2000). Inbreeding depression in conservation biology. Annu Rev Ecol Syst.

[4] Brook BW, Tonkyn DW, O’Grady JJ, Frankham R (2002). Contribution of inbreeding to extinction risk in threatened species. Conserv Ecol.

[5] Reed DH, Frankham R (2003). Correlation between fitness and genetic diversity. Conserv Biol.

[6] Liberg O, Andrén H, Pedersen H-C, Sand H, Sejberg D, Wabakken P (2005). Severe inbreeding depression in a wild wolf (*Canis lupus*) population. Biology Lett.

[7] Blomqvist D, Pauliny A, Larsson M, Flodin L-Å (2010). Trapped in the extinction vortex? Strong genetic effects in a declining vertebrate population. BMC Evol Biol.

[8] Feng S, Fang Q, Barnett R, Li C, Han S, Kuhlwilm M (2019). The genomic footprints of the fall and recovery of the Crested Ibis. Curr Biol.

[9] Segelbacher G, Cushman SA, Epperson BK, Fortin M-J, Francois O, Hardy OJ (2010). Application of landscape genetics in conservation biology: concepts and challenges. Conserv Genet.

[10] Frankham R (2015). Genetic rescue of small inbred populations: meta-analysis reveals large and consistent benefits of gene flow. Mol Ecol.

[11] Bowler DE, Benton TG (2005). Causes and consequences of animal dispersal strategies: relating individual behaviour to spatial dynamics. Biol Rev.

[12] Amos JN, Harrisson KA, Radford JQ, White M, Newell G, Nally RM (2014). Species- and sex-specific connectivity effects of habitat fragmentation in a suite of woodland birds. Ecology.

[13] Crochet P (2000). Genetic structure of avian populations—allozymes revisited. Mol Ecol.

[14] Lindsay DL, Barr KR, Lance RF, Tweddale SA, Hayden TJ, Leberg PL (2008). Habitat fragmentation and genetic diversity of an endangered, migratory songbird, the golden-cheeked warbler (*Dendroica chrysoparia*). Mol Ecol.

[15] Soikkeli M, Salo J (1979). The bird fauna of abandoned pastures. Ornis Fennica.

[16] Thorup O (1998). Ynglefuglene på Tipperne 1928–1992. Dansk Orn Foren Tidsskr.

[17] Thorup O. Breeding waders in Europe 2000. International Wader Studies 14. UK: International Wader Study Group; 2006. ISSN: 1354:9944.

[18] HELCOM (2013). Helcom Red List, Baltic marine environment protection commission—Helsinki Commission.

[19] Thorup O (1997). Langtidsstudier af Baltisk Ryle på Tipperne. Dansk Orn Foren Tidsskr.

[20] Pauliny A, Larsson M, Blomqvist D (2008). Nest predation management: effects on reproductive success in endangered shorebirds. J Wildlife Manage.

[21] Pakanen V-M, Luukkonen A, Koivula K (2011). Nest predation and trampling as management risks in grazed coastal meadows. Biodivers Conserv.

[22] Pakanen V-M, Thorup O (2016). Apparent adult survival of the critically endangered Baltic Dunlin *Calidris alpina schinzii* during a period of strong population decline. Bird Study.

[23] Soikkeli M (1970). Dispersal of dunlin *Calidris alpina* in relation to sites of birth and breeding. Ornis Fennica.

[24] Thorup O (1999). Breeding dispersal and site-fidelity in dunlin *Calidris alpina* at Tipperne. Denmark Dansk Orn Foren Tidsskr.

[25] Flodin L-Å, Blomqvist D (2012). Divorce and breeding dispersal in the dunlin *Calidris alpina*: support for the better option hypothesis?. Behaviour.

[26] Pakanen VM, Koivula K, Flodin L-Å, Grissot A, Hagstedt R, Larsson M (2017). Between-patch natal dispersal declines with increasing natal patch size and distance to other patches in the endangered Southern Dunlin *Calidris alpina schinzii*. Ibis.

[27] Wennerberg L, Marthinsen G, Lifjeld JT (2008). Conservation genetics and phylogeography of the Southern Dunlins *Calidris alpina schinzii*. J Avian Biol.

[28] Pakanen V-M, Aikio S, Luukkonen A, Koivula K (2016). Grazed wet meadows are sink habitats for the southern dunlin (*Calidris alpina schinzii*) due to nest trampling by cattle. Ecol Evol.

[29] Flodin L-Å, Larsson M, Ottvall R. Åtgärdsprogram för sydlig kärrsnäppa 2010–2014 (*Calidris alpina schinzii*). SEPA Report 6388. Stockholm: Swedish Environmental Protection Agency; 2010. ISBN: 978-91-620-6388-7.

[30] Ottvall R, Höglund J, Bensch S, Larsson K (2005). Population differentiation in the Redshank (*Tringa totanus*) as revealed by mitochondrial DNA and amplified fragment length polymorphism markers. Conserv Genet.

[31] Wenink PW, Baker AJ, Tilanus MGJ (1994). Mitochondrial control-region sequences in two shorebird species, the Turnstone and the Dunlin, and their utility in population genetic studies. Mol Biol Evol.

[32] Wennerberg L, Klaassen M, Lindström Å (2002). Geographical variation and population structure in the White-rumped Sandpiper *Calidris fuscicollis* as shown by morphology, mitochondrial DNA and carbon isotope ratios. Oecologia.

[33] Buehler D, Baker AJ (2005). Population divergence times and historical demography in Red Knots and Dunlins. Condor.

[34] Rönkä N, Kvist L, Pakanen V-M, Rönkä A, Degtyaryev V, Tomkovich P (2012). Phylogeography of the Temminck’s Stint (*Calidris temminckii*): historical vicariance but little present genetic structure in a regionally endangered Palearctic wader. Divers Distrib.

[35] Rönkä N, Pakanen V-M, Blomqvist D, Degtyarev VG, Golovatin M, Isakov GN (2019). Near panmixia at the distribution-wide scale but evidence of genetic differentiation in a geographically isolated population of the Terek Sandpiper *Xenus cinereu*s. Ibis.

[36] Küpper C, Edwards SV, Kosztolányi A, Alrashidi M, Burke T, Herrmann P (2012). High gene flow on a continental scale in the polyandrous Kentish Plover *Charadrius alexandrinus*. Mol Ecol.

[37] Thies L, Tomkovich P, dos Remedios N, Lislevand T, Pinchuk P, Wallander J (2018). Population and subspecies differentiation in a high latitude breeding wader, the Common Ringed Plover *Charadrius hiaticula*. Ardea.

[38] Marthinsen G, Wennerberg L, Lifjeld JT (2007). Phylogeography and subspecies taxonomy of Dunlins (*Calidris alpina*) in western Palearctic analysed by DNA microsatellites and amplified fragment length polymorphism markers. Biol J Linn Soc.

[39] Pakanen V-M, Jaakkonen T, Saarinen J, Rönkä N, Thomson RL, Koivula K (2018). Migration strategies of the Baltic Dunlin: rapid jump migration in the autumn but slower skipping type spring migration. J Avian Biol.

[40] Miller MP, Haig SM, Mullins TD, Ruan L, Casler B, Dondua A (2015). Intercontinental genetic structure and gene flow in Dunlin (*Calidris alpina*), a potential vector of avian influenza. Evol Appl.

[41] Schlaepfer MA, Runge MC, Sherman PW (2002). Ecological and evolutionary traps. Trends Ecol Evol.

[42] Thorup O (2018). Population sizes and trends of breeding meadow birds in Denmark. Wader Study.

[43] Cramp S, Simmons KEL (1983). The birds of the Western Palearctic.

[44] Stroud DA, Davidson NC, Haanstra A (2004). Status of migratory wader populations in Africa and western Eurasia in the 1990s. Int Wader Studies.

[45] Thorup O, Timonen S, Blomqvist D, Flodin L-Å, Jönsson PE, Larsson M (2009). Migration and wintering of Baltic Dunlins *Calidris alpina schinzii* with known breeding origin. Ardea.

[46] Wenink PW, Baker AJ, Rösner H-U, Tilanus MGJ (1996). Global mitochondrial DNA phylogeography of Holarctic breeding dunlins (*Calidris alpina*). Evolution.

[47] Wennerberg L, Bensch S. Geographic variation in the Dunlin *Calidris alpina* as revealed by morphology, mtDNA and microsatellites. In: Wennerberg L. Genetic variation and migration of waders. PhD thesis. Lund: University of Lund; 2001. p. 43–55. ISBN: 91-7105-161-9.

[48] Elts J, Leito A, Leivits A, Luigujõe L, Nellis R, Ots M (2019). Status and numbers of Estonian birds, 2013–2017. Hirundo.

[49] Naturvårdsverket. Uppdaterad åtgärdstabell för sydlig kärrsnäppa, 2016–2019 (*Calidris alpina schinzii*). Stockholm: Naturvårdsverket; 2018. http://www.naturvardsverket.se/Documents/publ-kompl/Uppdaterad-atgardstabell-sydlig-karrsnappa-6388-2018dec.pdf. Accessed 30 Mar 2020.

[50] Sambrook J, Russel DW (2001). Molecular cloning: a laboratory manual.

[51] van Oosterhout C, Hutchinson WF, Wills DPM, Shipley P (2004). MICROCHECKER: software for identifying and correcting genotyping errors in microsatellite data. Mol Ecol Notes.

[52] Raymond M, Rousset F (1995). GENEPOP (version 1.2.): population genetics software for exact tests and ecumenicism. J Hered.

[53] Rousset F (2008). Genepop’007: a complete re-implementation of the Genepop software for Windows and Linux. Mol Ecol Resour.

[54] Goudet J. FSTAT, a program to estimate and test gene diversities and fixation indices (version 2.9.3.2). 2002. Updated from Goudet J. FSTAT (Version 1.2): A computer program to calculate F-statistics. J Hered. 1995;86:6. http://www2.unil.ch/popgen/softwares/fstat.htm. Accessed Aug 2019.

[55] Amos W, Worthington J, Fullard K, Burg TM, Croxall JP, Bloch D (2001). The influence of parental relatedness on reproductive success. Proc R Soc Lond B.

[56] Excoffier L, Laval G, Schneider S (2005). ARLEQUIN ver 3.0.: an integrated software package for population genetics data analysis. Evol Bioinform.

[57] Hardy OJ, Vekemans X (2002). SPAGEDI: a versatile computer program to analyse spatial genetic structure at the individual or population levels. Mol Ecol Notes.

[58] Loiselle BA, Sork VL, Nason J, Graham C (1995). Spatial genetic structure of a tropical understory shrub, *Psychotria officinalis* (Rubiaceae). Am J Bot.

[59] Pons O, Petit RJ (1996). Measuring and testing genetic differentiation with ordered versus unordered alleles. Genetics.

[60] Slatkin M (1995). Measure of population subdivision based on microsatellite allele frequencies. Genetics.

[61] Pritchard JK, Stephens M, Donelly P (2000). Inference of population structure using multilocus genotype data. Genetics.

[62] Falush D, Stephens M, Pritchard JK (2003). Inference of population structure using multilocus genotype data: linked loci and correlated allele frequencies. Genetics.

[63] Hubisz MJ, Falush D, Stephens M, Pritchard JK (2009). Inferring weak population structure with the assistance of sample group information. Mol Ecol Resour.

[64] Puechmaillie SJ (2016). The program STRUCTURE does not reliably recover the correct population structure when sampling is uneven: sub-sampling and new estimators alleviate the problem. Mol Ecol Resour.

[65] Earl DA, von Holdt BM (2012). STRUCTURE HARVESTER: a website and program for visualizing STRUCTURE output and implementing the Evanno method. Conserv Genet Resour.

[66] Evanno G, Regnaut S, Goudet J (2005). Detecting the number of clusters of individuals using the software STRUCTURE: a simulation study. Mol Ecol.

[67] Jakobsson M, Rosenberg NA (2007). CLUMPP: a cluster matching and permutation program for dealing with label switching and multimodality in analysis of population structure. Bioinformatics.

[68] Jombart T (2008). *adegenet*: a R package for the multivariate analysis of genetic markers. Bioinformatics.

[69] Jombart T, Devillard S, Dufour A-B, Pontier D (2008). Revealing cryptic spatial patterns in genetic variability by a new multivariate method. Heredity.

[70] R Core Team. R: A language and environment for statistical computing. R Foundation for Statistical Computing, Vienna, Austria. 2018. https://www.R-project.org/. Accessed Aug 2019.

[71] Hedrick PW (1999). Perspective: highly variable loci and their interpretation in evolution and conservation. Evolution.

[72] Bird CE, Karl SA, Smouse PE, Toonen RJ (2011). Detecting and measuring genetic differentiation. Crustacean Iss.

[73] Kronholm I, Loudet O, de Meaux J (2010). Influence on mutation rate on estimators of genetic differentiation – lessons from *Arabidopsis thaliana*. BMC Genet.

[74] Meirmans PG, Hedrick PW (2011). Assessing population structure: F_ST_ and related measures. Mol Ecol Resour.

[75] Whitlock MC (2011). G’_ST_ and D do not replace F_ST_. Mol Ecol.

[76] Jost L (2008). G_ST_ and its relatives do not measure differentiation. Mol Ecol.

[77] Gerlach G, Jueterbock A, Kraemer P, Deppermann J, Harmand P (2010). Calculations of population differentiation based on GST and D: forget GST but not all of statistics!. Mol Ecol.

