# Supplementary material for: Genetic differentiation in an endangered and strongly philopatric, migrant shorebird
Source: BMC Ecol Evol. 2021 Jun 19;21:125. doi: 10.1186/s12862-021-01855-0 (PMC8214799; doi:10.1186/s12862-021-01855-0)
Supplement: Supplementary file 1 — Additional file 1: Table S1. Summary of microsatellites used in this study. [file 12862_2021_1855_MOESM1_ESM.docx]

**Additional file 1, Table S1.** Microsatellite loci used in this study and their characteristics.

| Locus | Fluorescent label | Repeat motif | Size range (bp) | Number of alleles | Annealing °C | Original species | Reference, accession number or primer sequences |
| --- | --- | --- | --- | --- | --- | --- | --- |
| Ruff1 | FAM | Di | 164–224 | 16 | 54 | Ruff, *Philomachus pugnax* | Thuman *et al*., 2002 |
| Ruff6 | NED | Tetra | 122–150 | 8 | 52 | Ruff, *Philomachus pugnax* | Thuman *et al*., 2002 |
| Pgt83 | FAM | Di | 156–176 | 11 | 55 | Knot, *Calidris canutus* | GeneBank accession number AY198173 |
| Cme1 | FAM | Di | 80–106 | 13 | 56 | Pectoral Sandpiper, *Calidris melanotos* | Carter & Kempenaers, 2007 |
| Cme2 | VIC | Di | 142–161 | 9 | 54 | Pectoral Sandpiper, *Calidris melanotos* | Carter & Kempenaers, 2007 |
| Cme6 | FAM | Di | 182–220 | 18 | 56 | Pectoral Sandpiper, *Calidris melanotos* | Carter & Kempenaers, 2007 |
| Calp2 | VIC | Di | 124–148 | 11 | 58 | Dunlin, *Calidris alpina* | Wennerberg & Bensch, 2001 |
| CAS009877 | FAM | Tetra | 87–127 | 11 | 56 | Dunlin, *Calidris alpina* | Blomqvist & Pauliny, unpublished |
| CAS011379 | FAM | Tetra | 157–197 | 12 | 56 | Dunlin, *Calidris alpina* | Blomqvist & Pauliny, unpublished |
| CAS012162 | FAM | Tri | 146–171 | 7 | 56 | Dunlin, *Calidris alpina* | Blomqvist & Pauliny, unpublished |
| CAS023374 | FAM | Tri | 209–230 | 6 | 56 | Dunlin, *Calidris alpina* | Blomqvist & Pauliny, unpublished |
| CAS026519 | FAM | Tetra | 238–258 | 6 | 56 | Dunlin, *Calidris alpina* | Blomqvist & Pauliny, unpublished |
