# Supplementary material for: Genetic differentiation in an endangered and strongly philopatric, migrant shorebird
Source: BMC Ecol Evol. 2021 Jun 19;21:125. doi: 10.1186/s12862-021-01855-0 (PMC8214799; doi:10.1186/s12862-021-01855-0)
Supplement: Supplementary file 2 — Additional file 2: Figure S1. ΔK values from the Structure analysis. Table S2. Mean LnPs and standard deviations for different K-values from program Structure Harvester. [file 12862_2021_1855_MOESM2_ESM.docx]

**Additional file 2**

**Figure S1.** ΔK values for K-values from 2 – 10 from 10 replicates of each K-value in program Structure, obtained with program Structure Harvester.


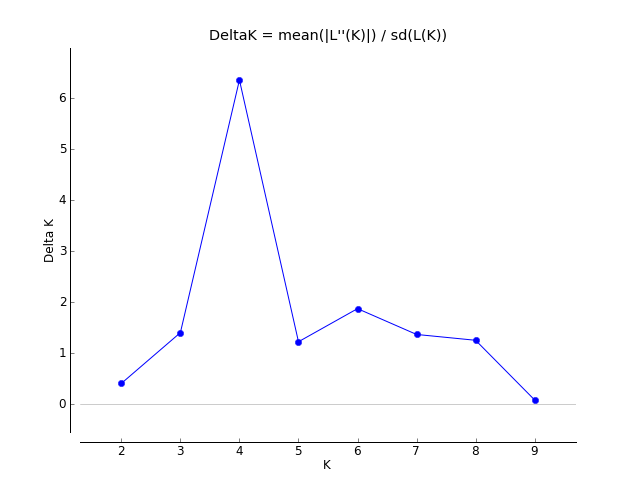


**Table S2.** Mean LnPs and standard deviations from 10 replicates in program Structure for K-values from 1-10, obtained with program Structure Harvester.

| # K | Reps | mean LnP | Standard deviation LnP |
| --- | --- | --- | --- |
| 1 | 10 | -7041.62 | 0.297396 |
| 2 | 10 | -6967.58 | 11.89153 |
| 3 | 10 | -6898.27 | 9.76047 |
| 4 | 10 | -6842.57 | 17.73847 |
| 5 | 10 | -6899.56 | 38.89502 |
| 6 | 10 | -7003.84 | 103.7828 |
| 7 | 10 | -7301.73 | 303.0121 |
| 8 | 10 | -7187.81 | 268.5404 |
| 9 | 10 | -7408.13 | 478.9038 |
| 10 | 10 | -7593.63 | 343.5396 |
