# Supplementary material for: Genetic differentiation in an endangered and strongly philopatric, migrant shorebird
Source: BMC Ecol Evol. 2021 Jun 19;21:125. doi: 10.1186/s12862-021-01855-0 (PMC8214799; doi:10.1186/s12862-021-01855-0)
Supplement: Supplementary file 3 — Additional file 3: Table S3. Information on locations of sampled populations, sampling years and permits. [file 12862_2021_1855_MOESM3_ESM.docx]

**Additional file 3, Table S3.** Locations of the sampled Baltic Southern Dunlin (*Calidris alpina schinzii*) and Dunlin (*C. a. alpina*) populations, sampling years and permit numbers (where applicable).

| Location | Coordinates | Sampling years | Permit number |
| --- | --- | --- | --- |
| *C. a. schinzii* |  |  |  |
| Finnish Bothnian Bay | 64°49'–65°4'N, 24°38'–25°16'E | 2009–2016 | PPO-2004-L-289-254; PPO-2006-L-206-254; POPELY/214/07.01.2013 |
| Pori, Finland | 61°3'N, 21°33'E | 2010–2011 | VARELY/324/07.01.2010 |
| Jurmo, Finland | 59°49'N, 21°35'E | 2010 | VARELY/324/07.01.2010 |
| Estonia | 58°16'–58°48'N, 23°2'–24°12'E | 2010–2012 | HLS14-4/19381-2 |
| Denmark | 55°52'N, 8°13'E | 2010–2013 |  |
| Western Sweden | 57°7'–57°55'N, 11°47'–12°8'E | 1997–2013 | 52/97, 106/99 (Göteborg), M 76-04 (Malmö/Lund) |
| Eastern Sweden | 56°12'N, 16°24'E | 2006–2008 | 52/97, 106/99 (Göteborg), M 76-04 (Malmö/Lund) |
| Gotland | 57°30'N, 18°27'E | 2002 |  |
| Southern Sweden | 55°29'N, 12°54'E | 2006–2010 | 52/97, 106/99 (Göteborg), M 76-04 (Malmö/Lund) |
|  |  |  |  |
| *C. a. alpina* |  |  |  |
| Finnish Lapland | 68°55'–69°11'N, 20°58'–21°25'E | 2010–2017 | VARELY/3622/2017 |
